# 2D Vermiculite Nanosheets Encapsulated in Hydrogel Electrolyte for Shuttle‐Free and Dendrite‐Inhibited Zinc‐Iodine Batteries

**DOI:** 10.1002/advs.78083

**Published:** 2026-09-28

**Authors:** Yunlong Zhou, Chuanliang Wei, Huanyu Yang, Bin Li, Bo Yang, Jie Ren, Cheng Ye, Maofeng Zhang

**Affiliations:** ^1^ School of Chemistry and Chemical Engineering Hefei University of Technology Hefei China; ^2^ Instrumental Analysis Center Hefei University of Technology Hefei China

**Keywords:** functional additive, hydrogel electrolyte, vermiculite nanosheets, zinc‐iodine batteries, zinc‐ion batteries

## Abstract

Aqueous zinc‐iodine (Zn–I_2_) batteries are a promising next‐generation energy storage technology due to their intrinsic safety, low cost, and high energy density. However, their application is blocked by the shuttle effect of polyiodide in the I_2_ cathode and dendrite formation/side reactions in the Zn metal anode. Here, a dual‐network hydrogel electrolyte consisting of polyacrylamide (PAM), chitosan (CS), and 2D vermiculite nanosheet (VN) is designed as PAM/CS/VN (PCV) to simultaneously address issues in the I_2_ cathode and Zn metal anode. The 3D PCV network and 2D VN synergistically suppress the shuttle effect through physical confinement and strong adsorption. Hydrophilic/zincophilic groups in PCV reduce free‐water activity and promote uniform Zn deposition, while electronegative VN sites coordinate Zn^2+^, lowering the desolvation barrier and inhibiting dendrite formation. Under the regulation of multifunctional PCV, Zn||Zn symmetric batteries work for over 1100 h at 5 mA cm^−2^ and Zn–I_2_ batteries achieve a capacity retention of 97.4% after 10 000 cycles at 2 A g^−1^. The pouch battery delivers a capacity retention of 89.3% after 400 cycles at 0.5 A g^−1^. This novel electrolyte and its effective additive offer valuable insights into enhancing the stable operation of Zn–I_2_ batteries.

## Graphical abstract

A dual‐network hydrogel was prepared via a one‐pot method, incorporating vermiculite nanosheets as an additive. The negatively charged vermiculite nanosheets not only coordinate with zinc ions to facilitate Zn plating/stripping and suppress Zn dendrite growth, but also adsorb polyiodides to inhibit the shuttle effect, which significantly improves the overall electrochemical performance and cycling stability of the zinc‐iodine battery system.

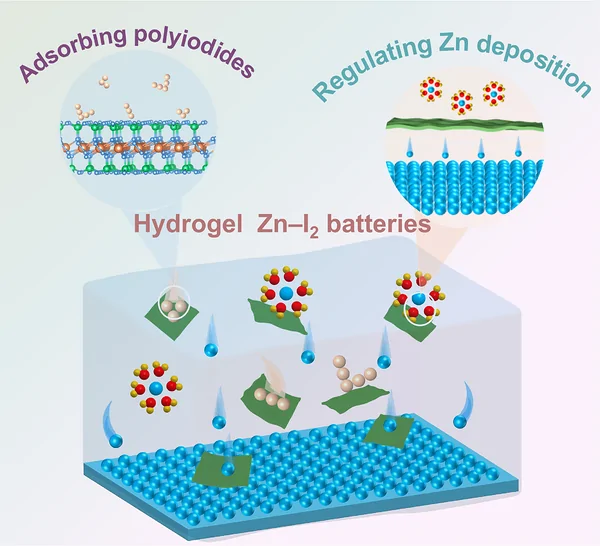

## 1 Introduction

Aqueous Zn–I_2_ batteries have demonstrated tremendous potential for large‐scale energy storage applications due to their safety/non‐toxicity, the abundance of zinc/iodine resources, and high theoretical specific capacity (211 mAh g^−1^) based on the I_2_/I^–^ redox couple [1, 2, 3, 4, 5]. Aqueous electrolytes with water as the solvent own high safety, excellent interfacial contact, and high ionic conductivity [6]. However, Zn–I_2_ batteries with aqueous electrolytes face a series of challenges (Figure 1a). On the cathode (I_2_) side, polyiodides can readily dissolve in aqueous electrolytes and shuttle to the Zn metal anode, leading to irreversible loss of active material, rapid capacity decay, and severe self‐discharge of batteries. More seriously, the shuttled polyiodides would chemically corrode the Zn metal anode and cause side reactions [7, 8]. On the anode (Zn) side, aqueous electrolytes can trigger the hydrogen evolution reaction (HER) and cause uneven Zn deposition and formation of Zn dendrites [9, 10]. These issues largely reduce the cycle life and stability of aqueous Zn–I_2_ batteries.

**FIGURE 1:**
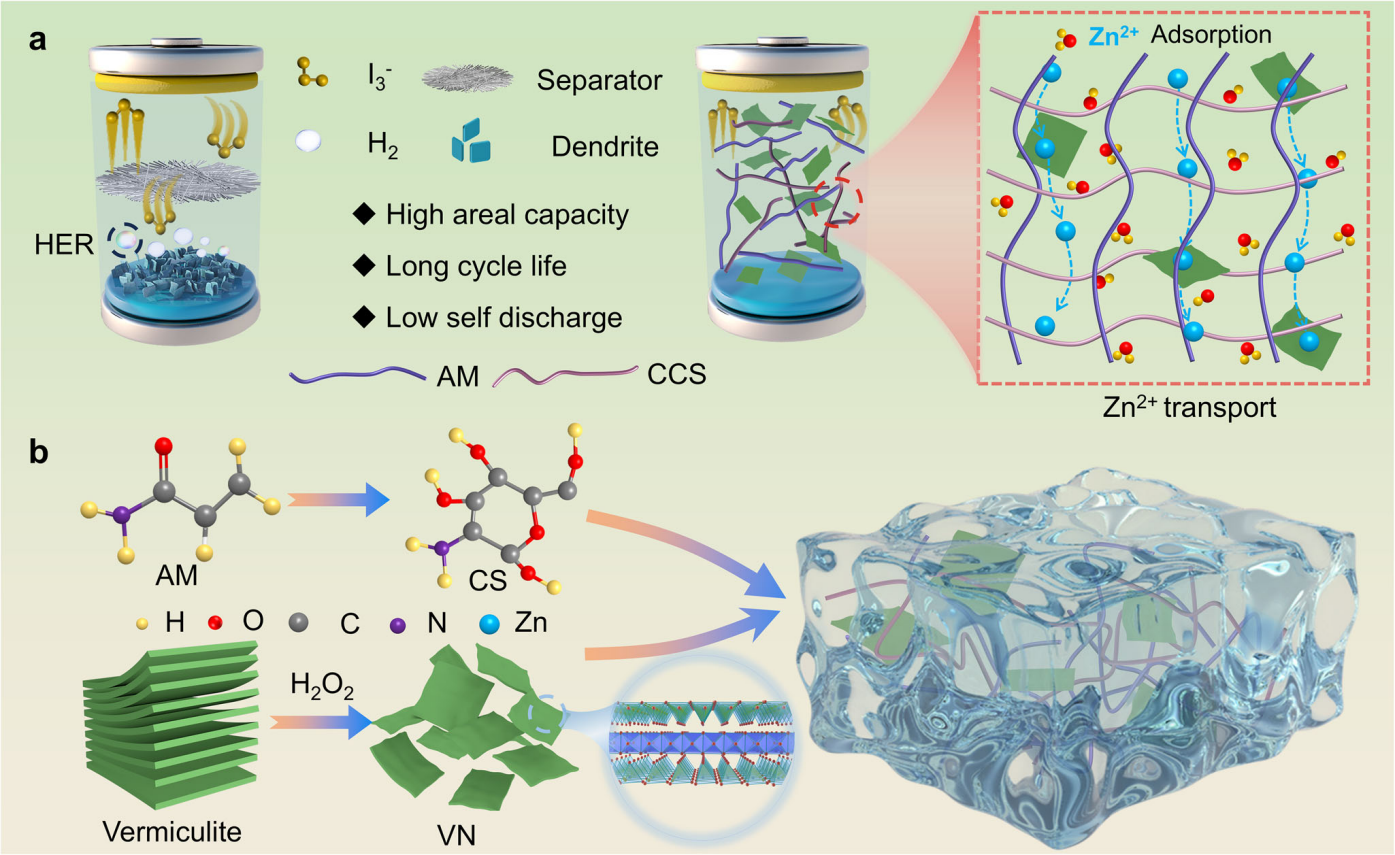
(a) Schematic illustration of the optimization of Zn–I_2_ battery with PCV. (b) Schematic illustration of the synthesis process of PCV.

Compared with aqueous electrolytes, hydrogel electrolytes are leakage‐free, flexible, and stretchable, which can inhibit dendrite growth, simplify device architecture, and offer both high ionic conductivity and safety [11]. Hydrogel electrolytes exhibit multiple advantages in Zn–I_2_ batteries due to their unique 3D cross‐linked network structure. The polar functional groups in hydrogels (─OH, ─NH_2_) can coordinate with Zn^2+^, regulate the distribution of ion flux, and guide Zn to deposit uniformly along favorable crystal planes ((002) plane), thereby inhibiting Zn dendrite growth [12, 13]. The 3D polymer network of hydrogels can physically confine polyiodide ions, reducing their migration to the Zn metal anode, thereby alleviating the shuttle effect and self‐discharge problems [14]. Despite significant progress in hydrogel electrolytes, several key challenges remain. Specifically, while cross‐linked polymer networks can confine polyiodide ions, they also impede the rapid transport of Zn^2+^ to some extent, leading to a lower ionic conductivity compared to liquid electrolytes [15, 16]. Moreover, the physical confinement effect of pure hydrogel networks on soluble polyiodide ions remains insufficient, and the shuttle effect persists over long‐term cycling [17, 18]. Therefore, the development of multifunctional and high‐performance hydrogel electrolytes is necessary.

Introducing functional additives into the hydrogel matrix is expected to further enhance its comprehensive performance without sacrificing the inherent advantages of the hydrogel [19]. For instance, Sun et al. [20] proposed (R)‐3‐aminobutyric acid (R‐3‐ABA) ‐filled integrated hydrogel electrolyte (RABA‐H), in which R‐3‐ABA self‐ionized to form R‐3‐ABA^+^ cations, which spontaneously adsorbed at the Zn anode interface to form an R‐3‐ABA^+^ cation interface layer. The R‐3‐ABA^+^ cation interface layer systematically regulated the multiphysical fields (concentration field, electric field, stress field) at the interface of the Zn metal anode, significantly improving the uniformity and reversibility of Zn deposition. The prepared Zn–I_2_ battery shows a capacity retention of 96.4% after 10 000 cycles at 2 A g^−1^. Wang et al. [21] proposed a hydrogel electrolyte by the combination of amidoxime‐functionalized porous polymer (AO‐PIM‐1) and sodium alginate (Alg). AO‐PIM‐1 was synthesized by the amidoxime reaction of the polymer PIM‐1 with an inherent microporous structure, featuring a rigid microporous skeleton and abundant hydrophilic ─NH_2_ and ─OH functional groups. Its microporous structure was conducive to the rapid transport of ions, while the amidoxime groups suppressed the shuttle effect of polyiodides through electrostatic repulsion. The assembled Zn–I_2_ full battery still maintained a high Coulombic efficiency (CE) of over 99.4% after 8700 cycles. However, most previously modification strategies of hydrogel electrolytes for Zn–I_2_ battery only achieve progressive improvements in a single performance indicator, making it difficult to balance the dual demands of Zn^2+^ transport optimization and polyiodide shuttle suppression [22, 23]. Few reports offer an integrated solution that can simultaneously address all interrelated defects without sacrificing ionic conductivity or introducing complex manufacturing steps [24, 25].

In recent years, 2D nanomaterials have received extensive attention due to their unique structures and excellent physical and chemical properties [26]. Vermiculite, as a natural layered silicate mineral, is composed of two silicon‐oxygen tetrahedral layers sandwiched between one magnesium‐oxygen octahedral layer, with exchangeable hydrated cations contained between the layers [27]. Vermiculite can be peeled layer by layer into single or few‐layer vermiculite nanosheets through physical or chemical exfoliation methods [28]. While the inherent chemical composition and crystal structure of vermiculite are preserved, the resulting VN exhibits a substantially reduced thickness and a markedly increased specific surface area. This exposes a greater number of surface‐active sites, thereby significantly enhancing interfacial interactions, ion‐exchange capacity, and adsorption performance [29, 30]. VN can be used as catalyst carriers and functional separator additives to enhance the dispersion and long‐term stability of active components [27, 31].

In this study, a hydrogel electrolyte composed of a rigid acrylamide (AM) network and a flexible CS network was designed through a one‐pot strategy, with 2D VN introduced as a multifunctional additive (Figure 1b). This hydrogel electrolyte, with its 3D cross‐linked network structure and abundant hydrophilic ─NH_2_ and ─OH functional groups, can form abundant hydrogen bonds with water molecules, thereby reducing water activity and facilitate Zn^2+^ coordination, providing rich channels for the transport of Zn^2+^. The 3D polymer network of the hydrogel inhibits the agglomeration of VN and buffers the volume changes during charging and discharging. At the same time, it can construct an efficient ion transport channel and improve the utilization rate of VN active sites. The excess negative charges on VN can facilitate the coordination of Zn^2+^ and reduce their desolvation energy barrier, thereby enhancing the plating/stripping kinetics and enabling dendrite‐free Zn deposition. In addition, the excellent capture ability of VN additives for polyiodide ions effectively inhibits the shuttling of polyiodide ions, thereby enhancing the utilization rate of redox‐active iodine substances and achieving efficient iodine conversion kinetics. Benefiting from these synergistic effects, the Zn||Zn symmetrical battery using PCV demonstrated an excellent cycling stability of more than 1100 h at 5 mA cm^−2^. The Zn–I_2_ battery with PCV electrolyte can provide a high reversible capacity of 145.5 mAh g^−1^ after 10 000 cycles at 2 A g^−1^, with a capacity retention of 97.4%.

## 2 Results and Discussion

Vermiculite exhibits a mica‐like layered structure, in which the basic structural unit consists of a magnesium octahedral layer sandwiched between two silica tetrahedral layers (Figure S1). Isomorphic substitution of Al^3+^ for Si^4+^ in the tetrahedral sheets generates a substantial net negative charge within these sheets [27]. The interlayer space contains water molecules and exchangeable cations (e.g., K^+^, Mg^2+^, Ca^2+^), imparting a high cation exchange capacity and thereby providing potential diffusion pathways for Zn^2+^ migration [28]. Since the stacked 2D layers of VN are bound solely by weak Van der Waals forces and hydrogen bonds, they can be readily exfoliated into single or few‐layer nanosheets [32]. In this work, we successfully prepared exfoliated VN with Zn^2+^ as the interlayer cation via H_2_O_2_‐assisted sonication combined with an ion‐exchange process (Figure 1b, Figure 2a) [30, 33]. Figure S2a shows the typical macroscopic morphology of the original vermiculite, presenting a golden‐brown color with a distinct lamellar stacked structure. Figure S2b shows that the volume of the VN has significantly expanded and has taken on a fluffy powder‐like form.

**FIGURE 2:**
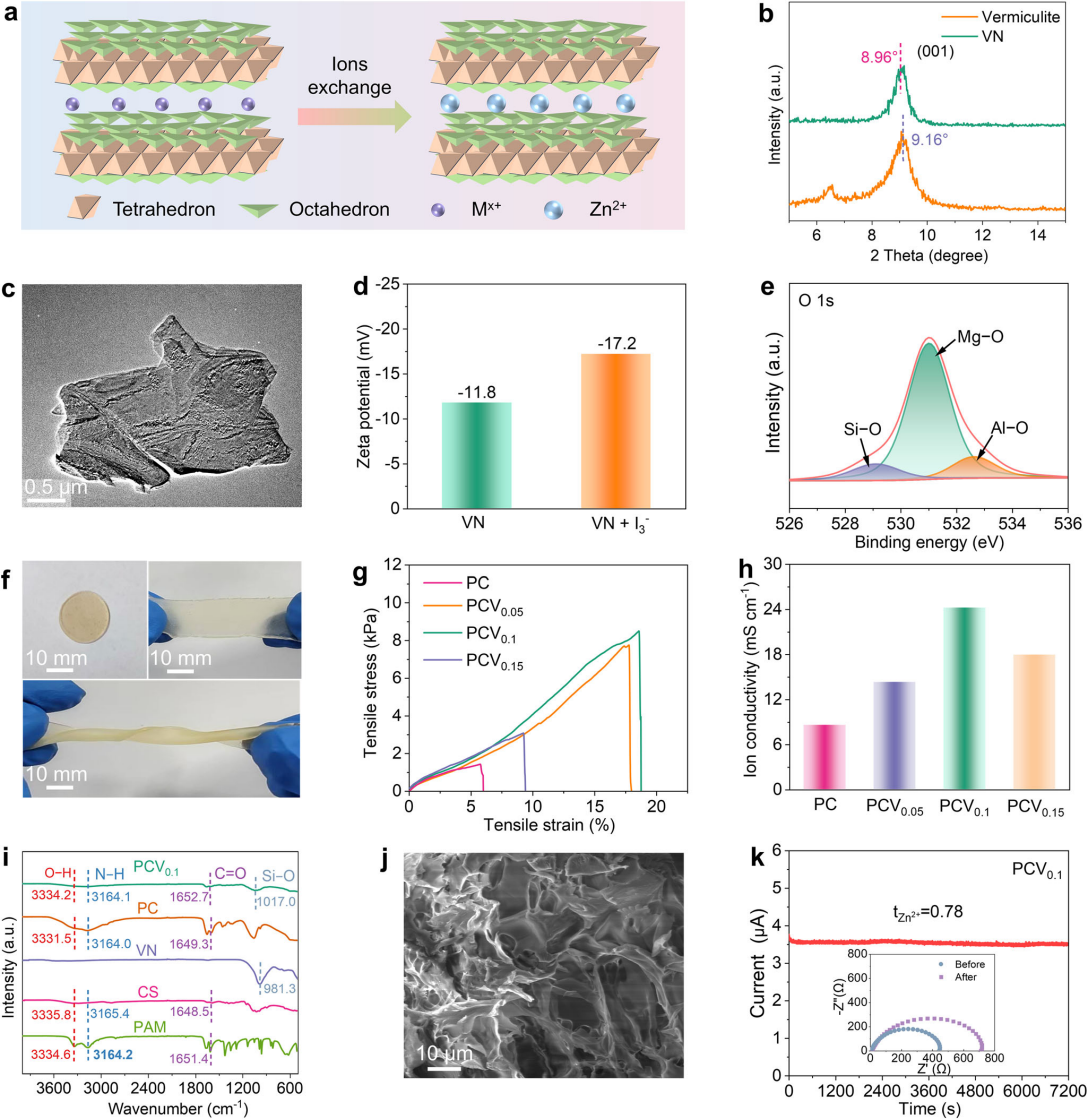
(a) Schematic diagrams of VN prepared via the Zn^2+^ exchange process. (b) XRD patterns of Vermiculite and VN. (c) TEM image of VN. (d) Zeta potential of VN and VN‐polyiodides complexs in aqueous solution. (e) O1s XPS spectra of the VN. (f) Optical images of PCV. (g) Tensile stress‐strain curves of hydrogel electrolytes with different VN contents. (h) The ionic conductivity of hydrogel electrolytes with different VN content. (i) FTIR spectra of PAM, CS, VN, PC and PCV_0.1_. (j) SEM image of freeze‐dried PCV_0.1_. (k) Transference number of Zn^2+^ for PCV_0.1_. EIS spectra of the Zn||Zn symmetric cell with PCV_0.1_ before and after polarization, inset: variation of current with time during polarization at an applied voltage of 10 mV at room temperature.

The shift of the interlayer peak to lower angles in the x‐ray diffraction (XRD) patterns of Zn^2+^‐exchanged VN further confirms the successful intercalation of Zn^2+^ into the interlayer region, resulting in an increased interlayer spacing (Figure 2b). Scanning electron microscopy (SEM) image of VN reveals its layered structure (Figure S3). Furthermore, as revealed by transmission electron microscopy (TEM), the exfoliated VN consists of approximately three or four layers and exhibits a typical nanoscale thickness (Figure 2c, Figure S4). O, Mg, Al, Si elements are detected on VN. VN can be stably suspended in a 2 M ZnSO_4_ aqueous solution, showing a distinct Tyndall effect (Figure S5). The zeta potential of VN in aqueous solution was measured to be −11.8 mV, indicating its potential adsorption capacity for Zn^2+^ (Figure 2d). Furthermore, the negative shift of the zeta potential (−17.2 mV) indicates that the polyiodides were effectively adsorbed on the VN surface. The x‐ray photoelectron spectroscopy (XPS) of VN (Figure S6a) shows characteristic peaks corresponding to O, Si, Al, Mg, and C elements. High‐resolution O1s spectra of VN identifies three distinct chemical bonds: Al─O (529.9 eV), Mg─O (531.6 eV), and Si─O (532.7 eV) (Figure 2e) [30]. High‐resolution Si 2p spectra show that the binding energy for the transition region from Si^2+^ to Si^4+^ in VN is 102.49 eV (Figure S6b). Compared to pure VN, PCV_0.1_ shows a positive Si 2p binding‑energy shift (from 102.04 to 102.11 eV), reflecting decreased Si─O electron density (Figure S7). This binding‑energy shift is attributed to hydrogen‑bonding or coordination interactions between the polar moieties (─NH_2_, ─OH, ─C═O) of PC and the surface Si─O groups of VN. These interactions withdraw electron density from the Si centers, consequently elevating their binding energy, in agreement with the FTIR blue shifts that signify strengthened bonds [34]. The negative interlayer charge of VN enables strong electrostatic adsorption of Zn^2+^. After encapsulating VN within a hydrogel, the interlayer spacing of VN can be effectively expanded or its interlayer microenvironment can be changed. In addition, the physical barrier effect of the polymer cross‐linked network structure jointly constitutes an effective inhibition of the shuttle effect of polyiodides.

A double network PCV polymer hydrogel was synthesized using flexible CS as the first network, rigid PAM as the second network, VN as an additive, and ZnSO_4_ as the electrolyte salt (Figure 1b). Figures S8 and S9 display digital images of the prepared PCV, which measures 5 × 5 cm and has a thickness of 0.7 mm. The polymer chain structure of PCV is illustrated in Figure S10, it features a dual‐network structure composed of polymerized AM molecular chains and CS molecular chains. In comparison, the main difference between PC and PCV lies in the absence of VN. Benefiting from this structure, the synthesized PCV hydrogel electrolyte can be arbitrarily cut, stretched, and twisted, exhibiting excellent mechanical flexibility and integrity (Figure 2f), which enables its potential application in wearable devices.

The mechanical properties of PCV hydrogel electrolytes with different VN contents (0.05, 0.1, and 0.15 wt.%, designated as PCV_0.05_, PCV_0.1_, and PCV_0.15_, respectively) were further evaluated. Tensile measurements (Figure 2g) indicate that as the VN content increases from 0 to 0.05 wt.% and 0.1 wt.%, both the tensile strain and tensile strength of the hydrogel electrolytes are improved. This can be attributed to the high Young's modulus of VN [32]. However, when the VN content increased to 0.15 wt.%, both tensile strain and strength decreased, which might be due to agglomeration caused by excessive addition of VN. Therefore, the PCV_0.1_ was determined to have the best mechanical properties. To study the migration kinetics of Zn^2+^, a Zn||Zn symmetric battery with PCV was constructed and its interface charge transfer impedance (Rct) was further tested. As shown in Figure 2h and Figure S11, when the VN content increases from 0.05 to 0.1 wt.%, the ionic conductivity significantly increases from 8.58 to 24.23 mS cm^−1^, which was superior to most previously reported hydrogel electrolytes (Table S1). As shown in Table S2, the water content of the PCV series hydrogels does not change much, but the ionic conductivity has significantly increased. This exceptionally high ionic conductivity can be attributed to the addition of VN, which provides high‐speed channels for Zn^2+^ transport [27]. Hydrogel electrolytes based on VN additives can stabilize the interface of Zn metal anode, reduce the concentration gradient of Zn^2+^ near the electrode, and accelerate the diffusion of Zn^2+^. However, further increasing the VN content to 0.15 wt.% leads to agglomeration due to excessive vermiculite addition, hindering ion transport and reducing the ionic conductivity to 17.95 mS cm^−1^. Due to van der Waals interactions, excessive loading of nano‐fillers can lead to agglomeration, thereby deteriorating the mechanical properties and ion/conductivity of the hydrogel [35, 36]. The charge transfer resistance (Rct) exhibits a similar pattern (Figure S12). The Rct values measured for Zn||Zn symmetric battery based on PC, PCV_0.05_, PCV_0.1_, and PCV_0.15_ are 589, 471, 225, and 356 Ω, respectively. The zinc‐philic property of vermiculite reduces the Zn diffusion energy barrier, leading to faster charge transfer and more efficient nucleation kinetics.

As shown in Figure 2i, the FTIR spectra of CS exhibit characteristic absorption bands at 3335.8, 3165.4, and 1648.5 cm^−1^, corresponding to the stretching vibrations of O─H, N─H, and C═O, respectively. For PAM, strong absorption peaks are observed at 3334.8, 3164.2, and 1651.4 cm^−1^, which also correspond to the stretching vibrations of O─H, N─H, and C═O [37]. For VN, a strong absorption peak at 981.3 cm^−1^ is attributed to the Si─O stretching vibration [30]. No new characteristic peaks were detected in the PC hydrogel. However, a new Si─O characteristic peak appears at 1017.0 cm^−1^ in PCV_0.1_, corresponding to the Si─O peak of VN. SEM of PCV_0.1_ shows that VN is cross‐linked with CS and PAM to form a continuous 3D network structure with interconnected pores (Figure 2j). The interconnected 3D pores not only enhance stress dissipation and prevent structural damage under deformation, but also promote the rapid transport of Zn^2+^, thereby improving mechanical robustness and ionic conductivity [38].

The Zn^2+^ transference numbers calculated by potentiostatic intermittent titration and EIS (Figure 2k, Figure S13) show that the Zn^2+^ transference numbers of liquid electrolyte (LE), PC, and PCV_0.1_ are 0.55, 0.58, and 0.78, respectively, reflecting that the addition of VN improves the ion channels, promoting ordered transport and uniform Zn deposition.

To further investigate the corrosion resistance and the inhibitory effect on HER, the self‐corrosion behavior of Zn in LE, PC, and PCV_0.1_ was examined using linear sweep voltammetry (LSV) (Figure 3a). Under the same current density, PCV_0.1_ exhibited the most negative hydrogen evolution potential and corrosion potential, indicating its superior ability to suppress HER compared to PC and LE, thereby effectively reducing the reactivity of water and mitigating parasitic hydrogen evolution. As shown by the Tafel analysis in Figure 3b, the PCV_0.1_ hydrogel electrolyte exhibits the lowest corrosion current density (0.062 mA cm^−2^) and the highest (most positive) corrosion potential (−0.014 V), indicating the combination of Zn with PCV_0.1_ significantly enhances the anti‐corrosion capability.

**FIGURE 3:**
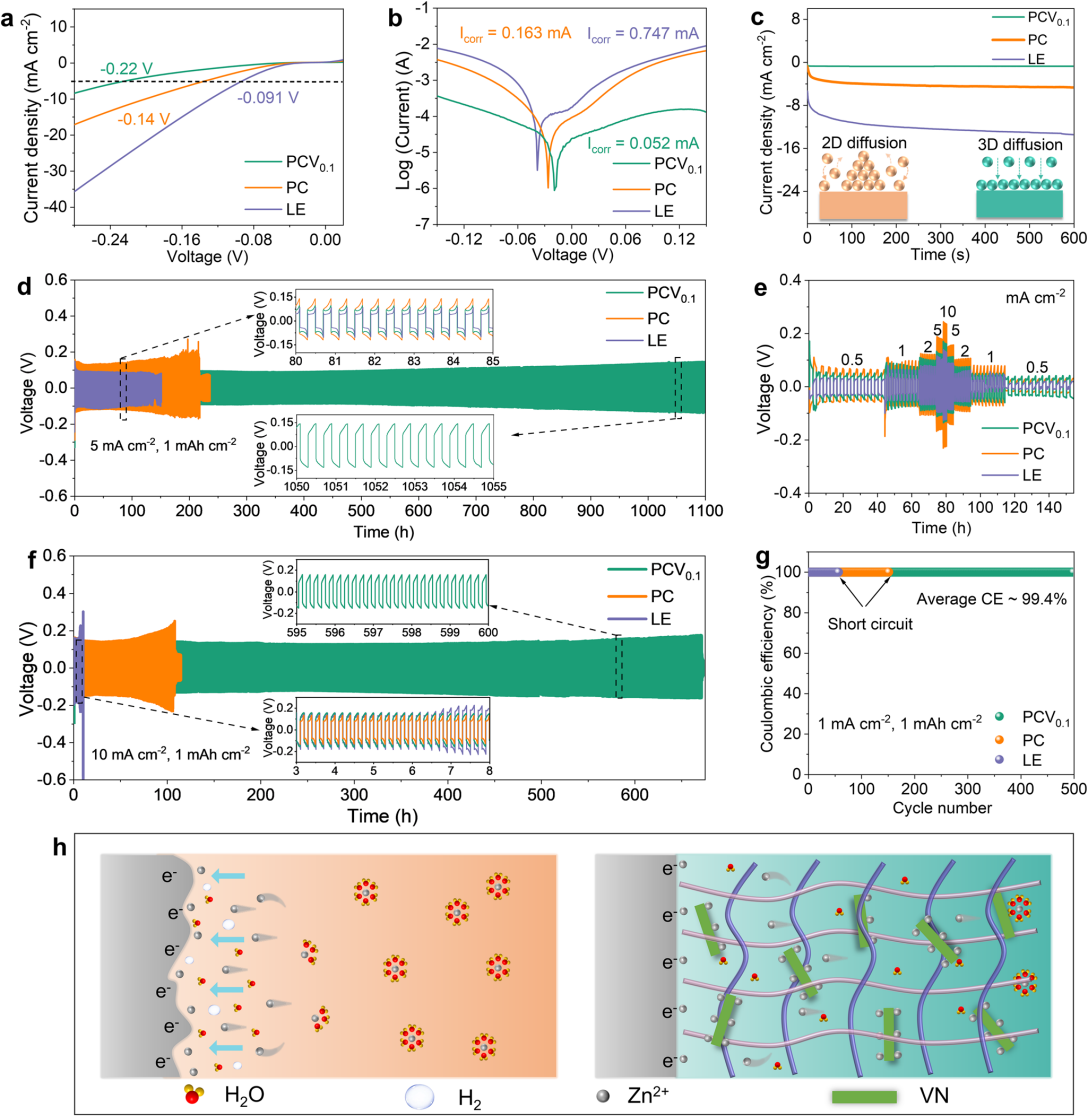
(a) LSV curves for different electrolytes. (b) Tafel curves for Zn||Zn symmetric batteries. (c) Chronoamperometry curves of Zn||Zn symmetric batteries under a constant voltage of −150 mV. (d) Cycling performance of Zn||Zn symmetric batteries with LE, PC and PCV_0.1_ electrolyte at 5 mA cm^−2^/1 mAh cm^−2^. (e) Rate performance of Zn||Zn symmetric batteries at different current densities. (f) Cycling performance of Zn||Zn symmetric batteries with LE, PC and PCV_0.1_ at 10 mA cm^−2^ /1 mAh cm^−2^. (g) CE of Zn||Cu asymmetric batteries at 1 mA cm^−2^/1 mAh cm^−2^. (h) The mechanism diagram of LE and PCV_0.1_ for promoting ion transfer and inhibiting Zn dendrite growth.

The nucleation behavior of Zn deposition was analyzed by chronoamperometry (CA) (Figure 3c). In battery with PCV_0.1_, the deposition mechanism transitioned from 2D diffusion to 3D diffusion within approximately 30 s. This shift facilitated the uniform nucleation of Zn, resulting in a smooth and dense Zn deposition layer. In contrast, the LE system demonstrated prolonged 2D diffusion behavior, characterized by a rapid current decrease during the first 100 s. This behavior led to preferential Zn deposition in localized high‐field regions, which in turn induced dendrite growth [39, 40].

The influence of PCV_0.1_ on interfacial reaction kinetics was evaluated. To assess the reversibility of the Zn plating/stripping process, cyclic voltammetry (CV) was performed on asymmetric Zn||Cu batteries (Figure S14). At a scan rate of 1 mV s^−1^, the Zn||Cu battery with PCV_0.1_ showed a lower nucleation overpotential than that with LE. This translates to an augmented thermodynamic driving force for Zn deposition within the PCV_0.1_ electrolyte, ultimately yielding a denser and highly uniform deposition morphology. As shown in Figure S15, PCV_0.1_ electrolyte exhibited a nucleation overpotential of 33.42 mV, calculated from the voltage gap between the nucleation and growth plateaus. This value is notably lower than the 90.25 mV recorded for the LE. A lower nucleation overpotential indicates both suppressed parasitic reactions and a decreased nucleation barrier, collectively promoting favorable Zn deposition kinetics [41].

Zn||Zn symmetrical batteries were assembled to explore the influence of different VN addition amounts on the stability of Zn metal anodes. The rate performance of the Zn||Zn symmetrical battery under each electrolyte system was tested at various current densities (0.5, 1, 2, 5, and 10 mA cm^−2^) (Figure S16). The battery with PCV_0.1_ exhibits a lower polarization voltage than batteries using PCV_0.05_ and PCV_0.15_, indicating PCV_0.1_ could enable a better reversibility and electrochemical stability for the Zn plating/stripping process. The Zn||Zn symmetrical battery with PCV_0.1_ maintained stable and long cycling performance for over 2600 h at 1 mA cm^−2^ and 1 mAh cm^−2^, which was much better than batteries with PCV_0.05_ and PCV_0.15_ (Figure S17). In addition, the cycling stability performance of hydrogel electrolytes based on PCV is stronger than that of VN directly suspended in liquid electrolytes. This is because direct suspension of VN in the LE would cause agglomeration within the separator. The above results indicated PCV_0.1_ is the best choice for batteries in this work. Based on these experimental results, a more detailed study of PVC_0.1_ is planned for subsequent experiments.

The effects of LE, PC and PCV_0.1_ on the stability of Zn metal anodes were explored. At 5 mA cm^−2^, the PCV_0.1_ system could demonstrate a stable operation of Zn||Zn symmetrical batteries for over 1100 h (Figure 3d), significantly outperforming PC (237 h) and LE (152 h). Zn||Zn symmetrical batteries with LE, PC and PCV_0.1_ were also tested at various current densities (0.5, 1, 2, 5, and 10 mA cm^−2^) (Figure 3e). At 0.5 mA cm^−2^, the plating/stripping curves of each electrolyte remain stable, but the overpotential of LE is the highest. As the current density increases from 0.5 to 10 mA cm^−2^, the polarization voltage rises accordingly. In contrast, the voltage increases of PCV_0.1_ is the smallest, reflecting its superior rate performance and interface stability. It can be also seen from Figure 3f that under a high current density of 10 mA cm^−2^, PCV_0.1_ can maintain a stable operation for 650 h. PCV_0.1_ can achieve a stable cycle of over 580 h at 5 mA cm^−2^ with a capacity of 5 mAh cm^−2^. While PC can only maintain for 110 h, and LE cannot even cycle normally (Figure S18). Compared with the hydrogel electrolytes previously reported in Table S3, the Zn||Zn symmetric battery with PCV_0.1_ demonstrated superior cycling performance. The above results highlight the significant effect of VN in inhibiting dendrite growth and parasitic reactions, thereby greatly enhancing the cycling stability and rate performance of Zn metal anodes.

To evaluate the effect of PCV_0.1_ on the Zn plating/stripping reversibility, Zn||Cu asymmetric batteries were tested at 1 mA cm^−2^ (Figure 3g) [42]. The PCV_0.1_‑based battery could stably cycle for over 500 cycles with an average CE of 99.4%, while the battery with LE failed after about 60 plating/stripping cycles due to dendrite‑induced short‑circuiting. Although PC extended the cycle life to 120 cycles, it still did not reach the level exhibited by PCV_0.1_. As shown in Figure S19, the Zn||Cu battery with PCV_0.1_ exhibits a stable voltage profile, with an initial CE of 94.26%, which is higher than batteries with PC (88.93%) and LE (83.16%). So PCV_0.1_ could enable a highly Zn plating/stripping process, indicating the effective suppression of side reactions by PCV_0.1_ during the early Zn deposition stage.

The regulation mechanism of Zn metal anode by PCV_0.1_ is proposed (Figure 3h). In the battery with LE, Zn^2+^ and H_2_O form a stable [Zn(H_2_O)_6_]^2+^ solvated structure. This structure is difficult to desolvate due to its high desorption energy, resulting in uneven diffusion of Zn^2+^ and subsequently inducing uncontrollable growth of Zn dendrites [38]. Meanwhile, [Zn(H_2_O)_6_]^2+^ desolvation releases a large number of active water molecules at the electrode/electrolyte interface, triggering side reactions such as hydrogen release, corrosion and passivation [14, 43]. In contrast, in batteries using PCV_0.1_ electrolyte, VN has a strong adsorption effect on Zn^2+^, which can lower the desolvation energy barrier and inhibit the HER [44]. In addition, VN provides a specific transport channel for Zn^2+^, which could promote uniform Zn deposition.

Morphological evolution of Zn metal anodes during the electrochemical process was monitored by SEM, confocal laser scanning microscopy (CLSM), and in situ optical microscopy. As shown in Figure 4a, the Zn metal anode cycled with LE displays a rough surface populated with numerous protruding and loosely packed Zn clusters, signifying a propensity for uncontrolled vertical growth and the subsequent evolution of dendrites. In contrast, Zn dendrites were significantly reduced on Zn metal anode in the battery with PC (Figure S20). However, Zn metal anode in the battery with PCV_0.1_ maintains a remarkably smooth and compact morphology, with no discernible by‐products or dendrite‐like structures (Figure 4b).

**FIGURE 4:**
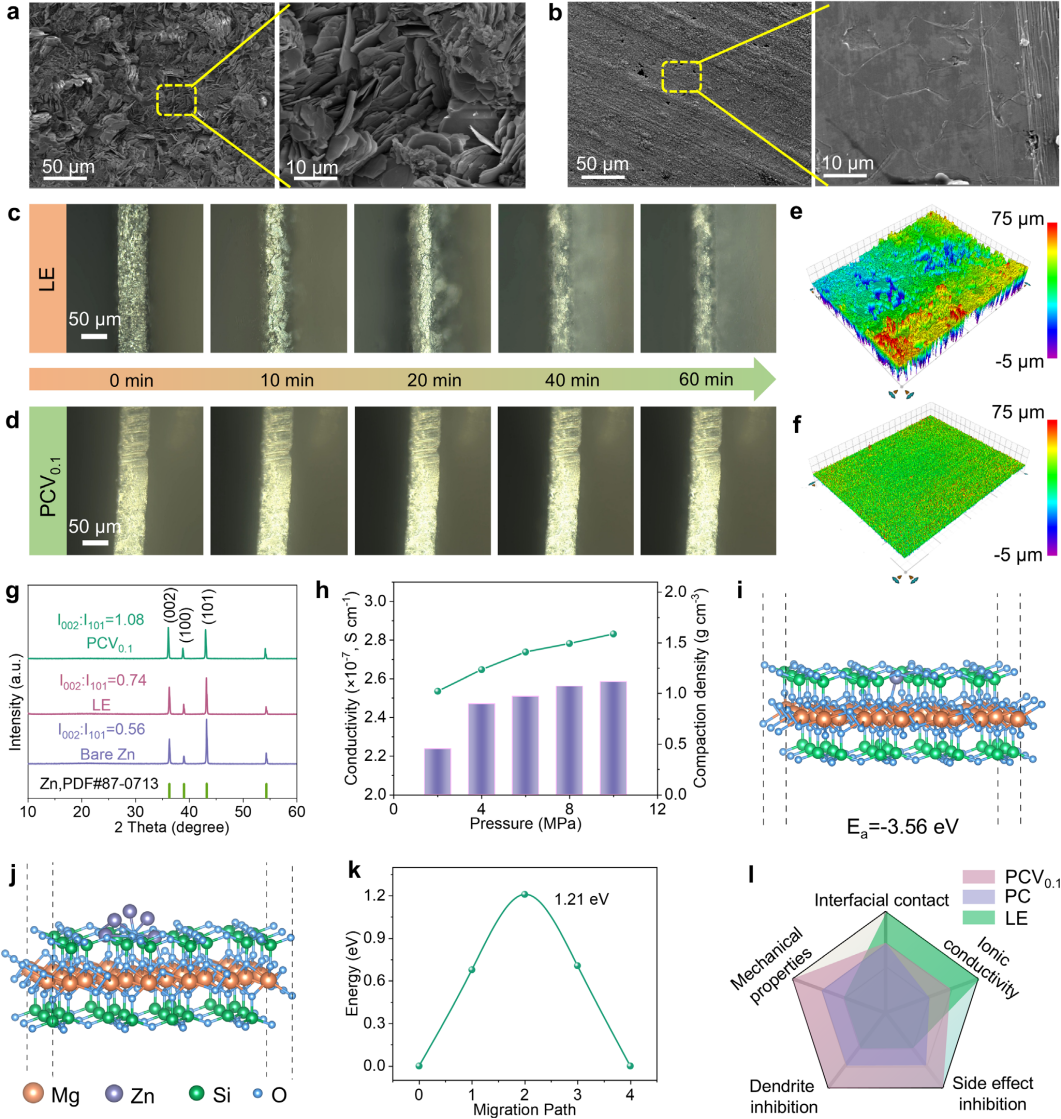
SEM images of Zn metal anode after 100 h of cycling in Zn||Zn symmetric batteries using (a) LE and (b) PCV_0.1_. In situ optical microscopy observations of Zn deposition progress at 10 mA cm^−2^ for 1 h with (c) LE and (d) PCV_0.1_. The corresponding CLSM images of Zn metal anode after 100 h of cycling with (e) LE and (f) PCV_0.1_. (g) XRD patterns of Zn metal anodes before and after 100 h of cycling with LE and PCV_0.1_. (h) Electrical conductivity VN at different pressures. The DFT‐calculated (i) binding energies of Zn atoms on VN. (j) Diffusion path of Zn atoms on VN and (k) corresponding calculated energy barrier. (l) Radar map of properties of different electrolytes.

In situ optical microscopy was used to directly observe the dynamic Zn deposition process [24]. As shown in Figure 4c, irregular point‐like deposits were observed on Zn metal anode in LE electrolyte, and they grew over time and form Zn dendrites. In contrast, the Zn surface modified with PCV_0.1_ displayed a uniform and smooth deposition, without obvious dendrite formation and by‐product accumulation, indicating that the Zn^2+^ flux remained more homogeneous during the deposition process (Figure 4d). The strong consistency between these results and the SEM images lead to the conclusion that PCV_0.1_ can effectively inhibit dendrite formation and stabilize the interface of Zn metal anode. The significant morphological disparity is further quantified by CLSM [45]. As shown in Figure 4e,f and Figure S21, the surface roughness of the Zn metal anode drops dramatically from 70.3 µm in the LE to merely 15.5 µm in the PCV_0.1_, indicating PCV_0.1_ can inhibit the growth of Zn dendrites and promote dense Zn deposition.

XRD was conducted to investigate the surface crystallographic evolution of Zn metal anode with LE and PCV_0.1_ after 100 h. The peak intensity ratio of the (002) plane to the (101) plane (I(002)/I(101)) was used to evaluate the preferred crystallographic orientation of the deposited Zn layer [21]. The I(002)/I(101) ratio of the Zn metal anode after cycling with PCV_0.1_ was measured to be 1.08 (Figure 4g), considerably higher than the value of 0.74 obtained with LE, suggesting a strong preferential orientation of Zn deposition along the (002) plane within the PCV_0.1_ system. Owing to the lowest surface energy of (002) plane, this orientation is favorable for inhibiting Zn dendrite growth. VN exhibits an ultralow electrical conductivity (Figure 4h). At pressures of 2, 4, 6, 8, and 10 MPa, the electrical conductivities of VN were measured to be 0.224, 0.247, 0.251, 0.256, and 0.258 µS cm^−1^, respectively. The ultralow electrical conductivity of VN avoids the risk of short circuits in batteries when using as an electrolyte additive [46].

Density functional theory (DFT) calculations were used to analyze the interaction between VN and Zn atoms [27]. The negative adsorption energy (−3.56 eV) of Zn^2+^ on the VN indicates a strong adsorption affinity of the VN toward Zn atoms (Figure 4i). The diffusion path (Figure 4j) and diffusion energy barrier of Zn atoms on VN were investigated by DFT (Figure 4k). The diffusion energy barrier was calculated to be 1.21 eV, which is lower than that of other materials that have been reported [47, 48, 49]. The results show that the presence of VN lowers the diffusion barrier for Zn ions and accelerates their transport across the electrode interface. Based on the above experimental data and theoretical calculation results, VN as a hydrogel additive has demonstrated significant advantages in inhibiting Zn dendrite growth and reducing side reactions (Figure 4l).

A Zn–I_2_ battery was assembled with activated carbon/iodine (AC@I_2_) as the cathode, Zn foil as the anode and PCV_0.1_ as the electrolyte (Figure S22). The electrochemical behaviors of Zn–I_2_ batteries were studied by CV between 0.6 and 1.8 V (vs. Zn/Zn^2+^) at scan rates varying from 0.5 to 5 mV s^−1^ (Figure 5a, Figure S23). As shown in Figure S24, the I_2_/I^−^ conversion process gave rise to only one reversible redox couple, and no redundant peaks corresponding to other intermediates were observed. This suggests that the intermediate polyiodides (I_3_^−^/I_5_^−^) undergo rapid and complete conversion, leaving no detectable residues. The contribution of capacitance was determined by fitting the scan‐rate dependence of the peak current using the equation i = a·v^b^, where i is the current, *v* is the scan rate, and *a* and *b* are adjustable parameters [50]. The results showed that the *b* values of the battery using PCV_0.1_ were 0.79 and 0.69 respectively, which were superior to those of PC (0.67/0.68) (Figure 5b; Figure S25), and reflected the enhanced surface control process. These higher *b* values reflect a greater contribution from surface‐controlled capacitive behavior, indicating enhanced reaction kinetics at the electrode surface [51]. The rate performance of Zn–I_2_ batteries containing different electrolytes was evaluated. Zn–I_2_ battery with PCV_0.1_ exhibited a better rate performance than that with PC (Figure 5c; Figure S26). The Zn–I_2_ battery with PCV_0.1_ achieved a discharge capacity of 203.6, 175.9, 162.9, 154.4 and 147.1 mAh g^−1^ at 0.1, 0.2, 0.5, 1, and 2 A g^−1^, respectively. The capacity recovered to 187 mAh g^−1^ when returning to 0.1 A g^−1^. In contrast, The Zn–I_2_ battery with PC achieved a discharge capacity of 181.7, 167.2, 153.3, 146.2, and 137.2 mAh g^−1^ at 0.1, 0.2, 0.5, 1, and 2 A g^−1^, respectively. The Zn–I_2_ battery with PCV_0.1_ also showed a better cycling performance. The Zn–I_2_ battery with PCV_0.1_ could deliver a capacity of 198.1 mAh g^−1^ after 500 cycles at 0.1 A g^−1^ (Figure 5d). In contrast, the battery using PC only showed a capacity of 186.2 mAh g^−1^ after 500 cycles, which was mainly attributable to the dissolution of polyiodides and the consequent shuttle effect. In the early stage of the cycle of PC‐based battery, the specific capacity of the battery shows a trend of first decreasing and then gradually recovering. This phenomenon can be attributed to the insufficient initial interface contact between the hydrogel electrolyte and the I_2_@AC cathode, which limits the utilization rate of iodine active sites. As the cycling process progresses, the hydrogel electrolyte undergoes slow creep under the weak pressure inside the battery and gradually penetrates into the multi‐level pore structure of AC, thereby exposing more iodine active sites for reversible redox reactions and promoting the gradual recovery and increase of capacity. Long‐term cycling test further demonstrated the stability of the PCV_0.1_‐based battery, which retained a capacity of 145.5 mAh g^−1^ after 10 000 cycles at 2 A g^−1^, representing a capacity retention of 97.4%. By contrast, the battery with PC exhibited a relatively poor performance, delivering only 118.7 mAh g^−1^ after 4100 cycles before undergoing a short circuit and ceasing to operate (Figure 5e, Figure S27). Furthermore, after 48 h of rest, the Zn–I_2_ containing PCV_0.1_ retained a self‐discharge efficiency of 82.90%, substantially outperforming the PC‐based batteries (73.53%) (Figure S28). The superior performance of PCV_0.1_‐based Zn–I_2_ batteries underscores the ability of VN to anchor polyiodides and prevent their migration to the Zn metal anode, thus effectively inhibiting self‐discharge and enhancing the overall electrochemical stability. Figure 5f summarizes the optimization mechanism of PCV_0.1_ for Zn–I_2_ batteries. VN with electronegativity can coordinate with Zn^2+^, thereby accelerating Zn^2+^ transport and inhibiting Zn dendrite growth [52]. In addition, due to the excellent adsorption and fixation effect of VN on polyiodide ions, it can promote the conversion of I^−^, I_3_^−^, and I_2_ on the cathode [30]. Due to the interaction between silica‐oxygen and iodide, polyiodide ions can be adsorbed on the surface of VN nanosheets suspended in the bulk electrolyte. Meanwhile, the hydrogel electrolyte exhibits a certain physical confinement effect on polyiodide ions due to its polymer chain segments. The introduction of VN further provides chemical adsorption anchoring sites, enhancing the capture capacity. The synergistic effect of the two enables the efficient capture of polyiodides in the positive electrode region and their rapid conversion into I^−^, thereby achieving the efficient and reversible utilization of iodine active substances. A comparative analysis of the relevant literature reveals that the PCV_0.1_ proposed in this work exhibits clear advantages, as summarized in Figure 5g and Table S4 [43, 44, 50, 53, 54, 55, 56]. To probe the potential application ability, Zn–I_2_ pouch batteries with PCV_0.1_ were assembled (Figure 5h; Figure S29). The pouch battery delivers an initial discharge capacity of 140.5 mAh g^−1^ with a capacity retention of 89.3% after 400 cycles at 0.5 A g^−1^ (Figure 5i). The pouch batteries with PCV_0.1_ can enable the LED to emit light normally (Figure 5j).

**FIGURE 5:**
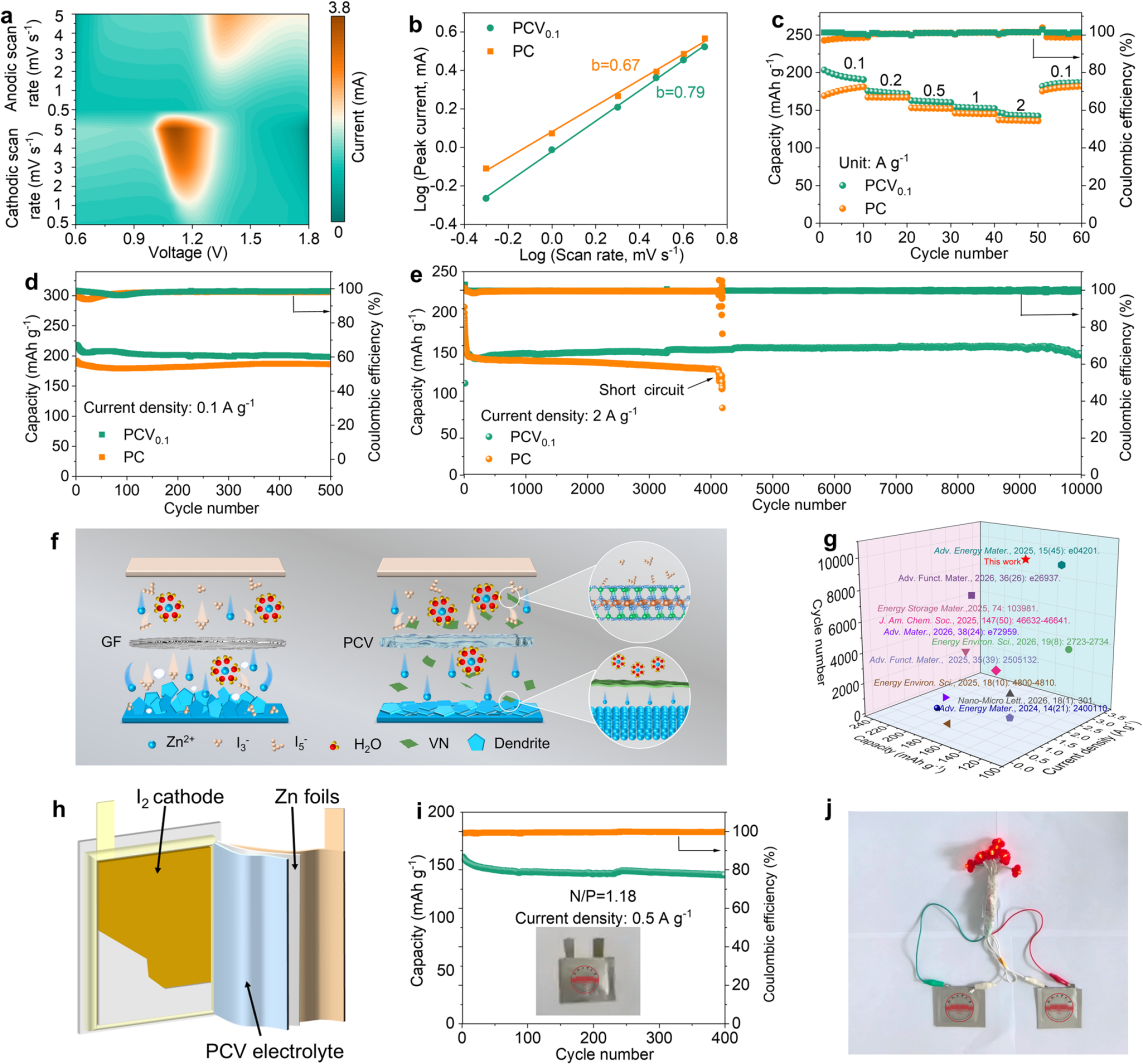
(a) CV contour plots of the Zn–I_2_ battery at different scanning rates. (b) Linear fitting of the oxidation and reduction peak currents against the square root of the scanning rate. (c) Rate performance of Zn–I_2_ battery with PC and PCV_0.1_. Cycle performance of Zn–I_2_ full batteries at (d) 0.1 A g^−1^ and (e) 2 A g^−1^. (f) Schematic diagram of optimization mechanism of PCV_0.1_ for Zn–I_2_ battery. (g) Comparison of electrochemical performance for Zn–I_2_ cells in this work with previous reports. (h) Schematic image of the Zn–I_2_ pouch battery. (i) Cycling performance of the Zn–I_2_ pouch battery. Inset: A photograph of a Zn–I_2_ pouch battery. (j) Photograph showing LED lights powered by pouch batteries.

To explore the adsorption mechanism of iodine species on the surface of VN, the interaction energies between VN and I_2__,_ I_3_^−^, and I_5_^−^ were calculated based on DFT (Figure 6a) [27]. The VN additive can form strong binding with the three‐iodine species (I_2_, I_3_^−^, and I_5_^−^), and the corresponding adsorption energies are −1.32, −3.28, and −1.28 eV respectively. The adsorption of iodine species by VN may result from the interaction between silicon‐oxygen bonds and iodine, similar to the adsorption of iodine by oxygen‐containing functional groups in carbon materials [27, 57]. In addition, iodine species can be effectively adsorbed on the surface of VN due to the unique electronegativity of the layered structure, the existence of surface silicon‐oxygen bonds, and the high 2D surface area. These results indicate that the addition of VN can effectively inhibit the shuttle effect of multiple iodide ions.

**FIGURE 6:**
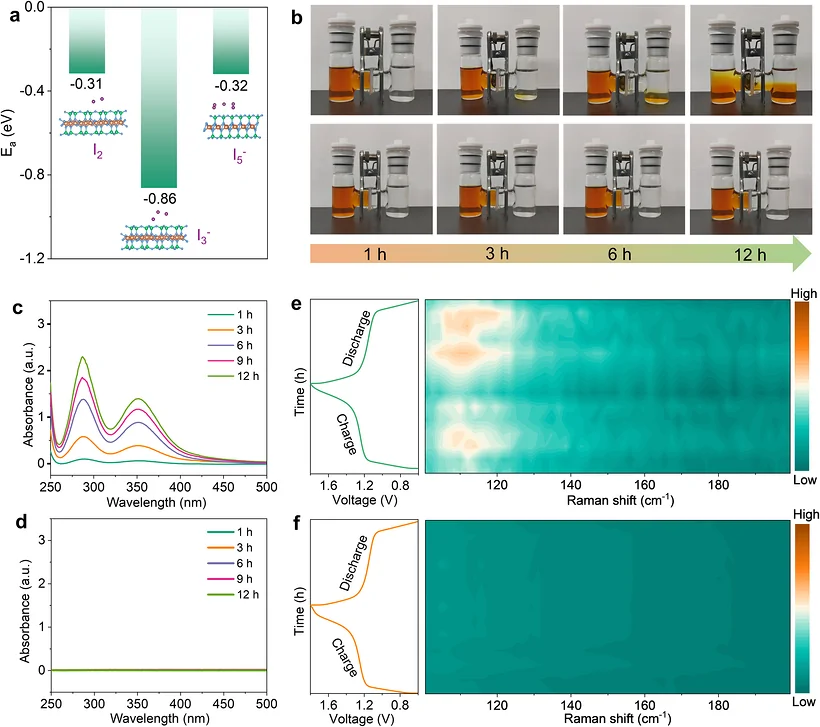
(a) The DFT‐calculated binding energies of iodide species on VN. (b) Optical images of the polyiodide solution in H‐type cell equipped with glass fiber and PCV_0.1_ electrolyte at various time intervals. UV–vis absorption spectra of the solution in the left chamber of the H‐type cell with (c) glass fiber separator and (d) PCV_0.1_. In situ Raman spectroscopy performed on Zn–I_2_ battery assembled with (e) glass fiber separator and (f) PCV_0.1_ to identify the evolution of polyiodide ions during the first charge–discharge process.

The shuttle behavior of polyiodides was investigated in H‐type electrolytic cells using glass fiber, PC and PCV_0.1_ as diaphragms respectively (Figure 6b) [58]. In the LE separated by a glass fiber diaphragm, the brownish‐yellow color of I_3_^−^ rapidly diffused to the left chamber within 1 h. After 12 h, the colors of the solutions on both sides were almost exactly the same, indicating a significant shuttle effect. In contrast, when PC was used, the left solution only showed a relatively light color change after 12 h, indicating the shuttle of iodine species was alleviated (Figure S30). Notably, PCV_0.1_ exhibited a more prominent capability to inhibit polyiodide shuttling, as evidenced by the absence of yellow discoloration in the right‐side solution even after 12 h, which indicates that VN incorporation can effectively adsorb iodine species.

The absorption signals of polyiodides in the right chamber of above H‐type electrolytic cells were quantified by ultraviolet‐visible (UV–vis) absorption spectroscopy [59]. UV–vis analysis confirmed that a peak for iodide absorption appeared in the battery with LE after 1 h, with a progressive intensification observed over the subsequent duration (Figure 6c). In contrast, for the PC system, this absorption peak does not appear until 3 h later, and its intensity is also significantly lower than that of the LE system (Figure S31). However, in the PCV_0.1_ system, no obvious absorption peak was observed (Figure 6d). The above experimental evidence fully confirms that VN with superior adsorption with polyiodide ions can efficiently inhibit their shuttling effect. This conclusion is consistent with the theoretical calculation results.

In‐situ Raman spectroscopy was utilized to evaluate the inhibitory performance of LE and PCV_0.1_ against polyiodide shuttling in Zn–I_2_ battery by monitoring the evolution of polyiodide species on Zn metal anode throughout the cycling process (Figure S32) [38, 60]. In Zn–I_2_ battery utilizing LE, the Raman spectra during the cycling process show two distinct bands at ≈110 cm^−1^, which are the characteristic peaks of I_3_^−^ (Figure 6e). In contrast, no obvious polyiodine signal was observed in Zn–I_2_ battery with PCV_0.1_ (Figure 6f). PCV_0.1_ exhibits superior confinement capability compared to LE, effectively suppressing the migration of polyiodides. Based on the above theoretical calculations and experimental results, the limiting effect of PCV_0.1_ on polyiodides has been confirmed. Such a limiting effect significantly mitigates the dissolution of active materials and curbs the shuttle effect, consequently advancing Zn–I_2_ battery toward superior capacity, extended cycle life, and minimized self‐discharge.

## 3 Conclusion

In summary, a multifunctional PCV hydrogel electrolyte incorporating VN additives is proposed to simultaneously suppress dendrite growth/corrosion at the Zn metal anode and polyiodide shuttling at the I_2_ cathode, enabling high‐performance Zn–I_2_ batteries. Hydrophilic and zincophilic functional groups (─OH, ─NH_2_) in the hydrogel reduce the activity of free water and facilitate uniform Zn deposition, which efficiently suppresses Zn dendrite propagation. The 3D polymer network of the hydrogel provides physical confinement for polyiodide ions and alleviates the shuttle effect. Electronegative VN can coordinate with Zn^2+^, lower the desolvation energy barrier of Zn^2+^, accelerate ion transport, and further inhibit Zn dendrite growth. Moreover, VN exhibits strong adsorption and immobilization capability toward polyiodides, which restricts their diffusion and migration, improves the utilization efficiency of iodine species, and optimizes the reaction kinetics of iodine conversion. Consequently, the Zn||Zn symmetric battery with the PCV_0.1_ hydrogel electrolyte achieves an ultralong cycling lifespan of over 1100 h at 5 mA cm^−2^ and 1 mAh cm^−2^. The assembled Zn–I_2_ battery maintains a high capacity retention of 97.4% after 10000 cycles at 2 A g^−1^. The dispersibility and catalytic activity of VN can be further enhanced, or advanced carbon materials with 3D hierarchical pores or heteroatom doping can be designed to strengthen iodine confinement and catalytic activity. Based on the fundamental mechanism of multifunctional electrolyte additives, this work offers a novel and feasible strategy for the development of advanced Zn–I_2_ batteries with high specific capacity and excellent long‐term cycling stability.

## Author Contributions

**Yunlong Zhou**: writing – original draft, writing – review and editing. **Chuanliang Wei**: writing – review and editing, formal analysis, supervision, funding acquisition. **Huanyu Yang**: writing – review and editing. **Bin Li**: writing – review and editing. **Bo Yang**: writing – review and editing. **Jie Ren**: writing – review and editing. **Cheng Ye**: investigation. **Maofeng Zhang**: writing – review and editing, supervision, formal analysis, funding acquisition.

## Conflicts of Interest

The authors declare no conflicts of interest.

## Supporting information




**Supporting File**: advs78083‐sup‐0001‐SuppMat.docx.

## Data Availability

The data that support the findings of this study are available in the supplementary material of this article.
