# Supplementary material for: 2D Vermiculite Nanosheets Encapsulated in Hydrogel Electrolyte for Shuttle‐Free and Dendrite‐Inhibited Zinc‐Iodine Batteries
Source: Adv Sci (Weinh). 2026 Sep 28:e78083. Online ahead of print. doi: 10.1002/advs.78083 (PMC13620259; doi:10.1002/advs.78083)
Supplement: Supplementary file 1 [file ADVS-9999-e78083-s001.docx]

Supporting Information

**2D Vermiculite Nanosheets Encapsulated in Hydrogel Electrolyte for Shuttle-Free and Dendrite-Inhibited Zinc–Iodine Batteries**

Yunlong Zhou^1^ | Chuanliang Wei^1^* | Huanyu Yang^1^ | Bin Li^1^ | Bo Yang^1^ | Jie Ren^1^ | Cheng Ye^2^ | Maofeng Zhang^1^*

^1^School of Chemistry and Chemical Engineering, Hefei University of Technology, Hefei 230009, China | ^2^Instrumental Analysis Center, Hefei University of Technology, Hefei 230009, China

Correspondence: Maofeng Zhang (mfzhang@hfut.edu.cn) | Chuanliang Wei ([clwei@hfut.edu.cn](mailto:clwei@hfut.edu.cn))

**Experimental section**

**Materials**

Acrylamide (AM, 99%), Zinc Sulfate (ZnSO_4_, 99%), N,N’-Methylenebisacrylamide (MBAA, 99%)，Iodine powder (I_2_, 99%), Activated carbon (AC, 99%), Polyvinylidene fluoride (PVDF) and N-methylpyrrolidone (NMP) were purchased from Aladdin Reagent Co. Ltd. Ammonium per sulfate (APS, 99%) Chitosan Zinc (CS, 99%) was purchased from Sinopharm Chemical Reagent Co., Ltd. Zn foil, copper foil and titanium foil are purchased online.

**Preparation of Vermiculite Nanosheets**

Vermiculite nanosheets were prepared by H_2_O_2_ ultrasonic exfoliation and ion exchange method. Vermiculite samples were added to 100 mL of H_2_O_2_ (30% wt) and treated for 15 hours. Then, the obtained samples were added to 1 M ZnSO_4_ solution and stirred at high speed for 3 days to obtain a monolayer colloidal suspension. The samples were washed three times and dried to obtain vermiculite nanosheets.

**Preparation of PCV**

0.1 g of chitosan (CS), 1.5 g of acrylamide (AM), 10 mg of N,N'-methylenebisacrylamide (MBAA), 9 mg of ammonium persulfate (APS), and various mass ratios of vermiculite nanosheets (VN) (0 wt%, 0.05 wt%, 0.1 wt%, and 0.15 wt%) were added to 4 mL of 2 M ZnSO_4_ solution under stirring. After all solids were completely dissolved, the mixture was poured into a mold (length: 8 cm, width: 8 cm, depth: 0.7 mm) and subjected to thermal polymerization at 60 °C for 2 hours to obtain PCV hydrogels with different vermiculite doping ratios.

**Preparation of I_2_ cathode**

The iodine powder was ground and mixed with activated carbon (AC) at a mass ratio of 1:1 for 5 minutes. The mixture was sealed in a glass vial and heated at 90 °C for 6 hours. Titanium foil was used as the current collector, the above I_2_/AC mixture as the active material, acetylene black as the conductive additive, and PVDF as the binder. The components were weighed at a mass ratio of 8:1:1, placed in a mortar, and mixed with an appropriate amount of NMP by grinding for 6 hours to obtain a homogeneous slurry. The slurry was coated onto a collector using a spatula and dried in a vacuum oven at 45 °C for 6 hours to ensure complete removal of the organic solvent. Finally, the dried film was pressed into circular electrodes with a diameter of 1.0 cm for further use. The mass loading of iodine was approximately 2.8–3.5 mg cm^-2^.

**The assembly of different batteries**

Commercial zinc foil (100 microns) is sanded on the surface of the zinc foil with 1200 Cw sandpaper for 5 minutes to give it a metallic luster. Immerse the polished metallic zinc in alcohol, ultrasonically clean it for 5 minutes, and then dry it with degreased cotton wool. Subsequently, cut the zinc foil into circular electrode sheets with a diameter of 1.0 cm and set them aside. The Zn||Zn symmetrical cell, Zn||Cu (10 um thick) half-cell and Zn||I_2_ full cell were assembled into the CR-2032 type coin cell and tested using the Lanhe (LAND) Battery Testing System. For batteries with liquid electrolyte (2 M ZnSO_4_), glass fiber (GF) is used as the separator, and the volume of the liquid electrolyte is 110 μL. For batteries using PCV gel electrolyte, the gel is cut into circular films with a diameter of 1.9 cm as the electrolyte, and then the battery is assembled together with the casing (CR-2032) and gasket to form a coin battery. Let the battery stand for 4 hours for subsequent electrochemical measurements. The pouch cells were assembled in ambient air using Al-laminated plastic film as the packaging material. The I_2_/AC active material was coated on a 5 cm × 6 cm Ti foil with an I_2_ loading of approximately 5 mg cm^−2^. The capacity ratio of anode to cathode (N/P ratio) was controlled at 1.18:1. The entire assembly process is completed in room-temperature air.

**Materials characterizations.**

The surface morphology of the circulating Zn electrode and the morphology of the hydrogel electrolyte were observed using field emission scanning electron microscopy (SEM). The tensile properties of the hydrogels were tested using an electronic universal testing machine. Dumbbell-shaped hydrogels (17 mm × 4 mm × 2 mm) were subjected to tensile tests at a speed of 100 mm min^-1^ and a load of 100 N. FTIR was performed for infrared characterization of the gel electrolytes on a Bruker TENSOR II infrared spectrometer in the wavelength range of 400-4000 cm^-1^. X-ray diffraction (XRD, Rigaku SmartLab SE) was carried out with pole piece scanning in the range of 5°~90°. X-ray photoelectron spectroscopy (XPS, Thermo Scientific K-Alpha) was used to test the elemental composition and chemical valence states of the gel electrolytes.

**Electrochemical measurements.**

The battery was subjected to constant current charge and discharge (GCD) tests using the Lanhe (LAND) Battery Testing System. Electrochemical cyclic voltammetry (CV), impedance spectroscopy (EIS), linear scanning voltammetry (LSV), and timed voltammetry (CA) tests were conducted on the CHI660E electrochemical workstation. CV tests were conducted on Zn||I_2_ batteries within the voltage range of 0.6 V to 1.8 V with a scanning rate of 0.1mV s^-1^. The Tafel test was conducted within the voltage range of -0.3 V to 0.3 V, with a scanning rate of 1 mV s^-1^. The LSV test adopts a dual-electrode system with a scanning rate of 1 mV s^-1^. The working electrode is stainless steel and the reverse electrode is zinc metal. CA tests were conducted on Zn||Zn symmetrical cells with an applied voltage of -150 mV and a running time of 600 seconds to observe the nucleation growth. The I-t test for transferring the quantity of Zn^2+^ was conducted under the Zn symmetrical battery configuration of 7200s, followed by another EIS test. The self-discharge test process is as follows: Firstly, the battery is subjected to 10 charge-discharge cycles at A current density of 1 A g^−1^, and its initial discharge capacity is recorded and denoted as C_0_. Subsequently, charge the battery at a constant current to the set upper limit cut-off voltage, and leave it in an open-circuit state for 48 hours. Record the remaining capacity and denote it as C_1_. The capacity retention rate is calculated by the formula C_1_/C_0_ × 100%.

**Calculation methods**

First principles calculations. All calculations in this study were performed with the Vienna ab initio Simulation Package (VASP) within the frame of density functional theory (DFT) [1]. The exchange-correlation interactions of electron were described via the generalized gradient approximation (GGA) with PBE functional [2], and the projector augmented wave (PAW) method [3] was used to describe the interactions of electron and ion. Additionally, the DFT-D3 method was used to account for the long-range van der Waals forces present within the system [4,5]. The Monkhorst-Pack scheme was used for the integration in the irreducible Brillouin zone. The kinetic energy cut-off of 450 eV was chosen for the plane wave expansion. The lattice parameters and ionic position were fully relaxed, and the total energy was converged within 10^-5^ eV per formula unit. The final forces on all ions are less than 0.02/Å. The energy barrier of ion migration was calculated use the CI-NEB method.

**Figure S1**. Schematic diagram of crystal structure of vermiculite.

**Figure S2**. (a) digital photo of the original vermiculite and (b) VN powder.

**Figure S3**. SEM image of VN.

**Figure** **S4**. TEM image of VN and the corresponding elemental mapping images of O, Mg, Al, and Si.

**Figure** **S5**. Tyndall effect of the ZnSO_4_-VN.

**Figure S6**. (a) XPS survey spectra and (b) Si 2p XPS spectra of VN.

**Figure S7**. Si2p XPS spectra of PCV_0.1._

**Figure S8**. The digital image of PCV_0.1_.

**Figure S9**. Photograph of measuring the thickness of the PCV_0.1_.

**Figure S10**. Polymer chains of (a) PAM and (b) CS.

**Figure S11**. (a) Schematic diagram of the conductivity testing setup and (b) Nyquist plots with different electrolytes.

The hydrogel sample was cut into a rectangle, and its dimensions were precisely measured with a vernier caliper. The effective contact area was directly calculated according to Equation 1:

$S=L\times W$	(Equation 1)

where L and W are the length and width respectively.

The ionic conductivity of the hydrogel electrolyte was calculated according to Equation 2:

$\delta=\frac{L}{R_{s}A}$ (Equation 2)

where L, R_s_, and A represent the thickness, resistance, and area of the separators, respectively. L represents the thickness of the hydrogel electrolyte, and S represents the contact area between the stainless steel plate and the hydrogel.

**Figure S12**. EIS curves of the Zn||Zn batteries with PCV_0_, PCV_0.05_, PCV_0.10_ and PC.

**Figure S13**. I-t curve of symmetric cells with (a) LE, and (b) PC electrolytes.

The insets are the corresponding EIS spectra before and after polarization. The Zn^2+^ transference number (*t_Zn2+_*) are calculated using the following equation 3:

$\text{t}\text{=}\frac{\text{I}_{\text{s}}\text{(∆}\text{V}\text{-}\text{I}_{\text{0}}\text{R}_{\text{0}}\text{)}}{\text{I}_{\text{0}}\text{(∆}\text{V}\text{-}\text{I}_{\text{s}}\text{R}_{\text{s}}\text{)}}$	(Equation 3)

where *R_0_* is the resistance before polarization, *R_s_* is resistance after polarization, *∆V* is the polarization potential, *I_0_* is the initial current, and *I_s_* is the stable state current.

**Figure S14**. Cyclic voltammogram of Zn nucleation on copper foil.

**Figure S15**. Nucleation overpotential curves for Zn||Cu batteries.

**Figure S16**. Rate performance of Zn||Zn symmetric batteries with gel electrolytes containing different VN concentrations.

**Figure S17**. Cycling performance of Zn||Zn symmetric batteries using PCV electrolytes with different VN contents and VN directly suspended in LE at 1 mA cm^−2^, 1 mAh cm^−2^.

**Figure S18**. Cycling performance of Zn||Zn batteries at 5 mA cm^−2^, 5 mAh cm^−2^.

**Figure S19**. Galvanostatic charge/discharge curves of Zn||Cu cells after different cycles in LE, PC and PCV_0.1_ under 1 mA cm^−2^, 1 mAh cm^−2^.

**Figure S20**. SEM images of Zn foils after 100 h cycling (a) with LE and (b) with PC electrolyte.

**Figure S21.** The roughness distribution curves of Zu electrodes after plating in the (a,b) PC and (c,d) PCV_0.1_.

**Figure S22**. Model of Zn||I_2_ battery.

**Figure S23**. CV curves of Zn‖I_2_ batteries with (a) PCV_0.1_ and (b) PC at different scan rate of 0.5 mV s^−1^ to 5 mV s^−1^.

**Figure S24.** CV curves of Zn||I_2_ batteries using different electrolyte at a scan rate of 5 mV s^−1^.

**Figure S25.** The corresponding linear relationships between log (peak current) and log (scan rate) for different electrolytes.

**Figure S26**. GCD curves of the Zn||I_2_ batteries with PCV_0.1_ at different current densities.

**Figure S27**. The GCD curves of the Zn||I_2_ battery with PCV_0.1_ under a current density of 2 A g^−1^.

**Figure S28**. Self-discharge test curves of AZIBs using (a) PCV_0.1_ and (b) PC electrolyte.

**Figure S29**. Digital images of Zn||I_2_ pouch cells.

**Figure S30**. Optical images of the polyiodide solution in H-type batteries equipped with PC electrolyte separators at various time intervals.

**Figure S31**. UV–vis absorption spectra of the solution in the left chamber of the H-type battery with PC electrolyte

\

**Figure S32**. Schematic diagram of the in-situ Raman spectroscopy measurement.

**Table S1.** Conductivities of gel electrolytes in reported works.

| Electrolyte | Conductivity (mS cm^-1^) | Reference |
| --- | --- | --- |
| PZG | 17.51 | [6] |
| CMC/SA-Zn^2+^ | 2.08 | [7] |
| CCH | 9.7 | [8] |
| CD-PEO/PAM | 22.4 | [9] |
| PAMPS-Zn | 15.6 | [10] |
| AO-PIM-1/Alg | 18.6 | [11] |
| PC-PVA/ZnCF_3_SO_3_ | 18.87 | [12] |
| PAM/SL/SA | 21.43 | [13] |
| SPAM/Gly | 17.2 | [14] |
| MNPHE | 16.8 | [15] |
| PPTA | 10.8 | [16] |
| PVA/ Bmim[ZnBr_3_] | 0.739 | [17] |
| This work | 24.23 |  |

**Table S2**. Water Content and ionic Conductivity of PCV Series Hydrogels

| Sample | Moisture content (wt%) | Ionic conductivity (mS cm^−1^) |
| --- | --- | --- |
| PC | 57.88 | 8.58 |
| PCV0.05 | 57.85 | 14.32 |
| PCV0.1 | 57.82 | 24.23 |
| PCV0.15 | 57.80 | 17.95 |

**Table S3.** Cycling performance of the Zn symmetrical cells with reported hydrogel electrolytes.

| Electrolyte | Current density | Cycle life (h) | Reference |
| --- | --- | --- | --- |
| PZG | 1.0 mA cm^−2^/1.0 mAh cm^−2^ | 2100 | [6] |
| AO-PIM-1/Alg | 1.0 mA cm^−2^/0.5 mAh cm^−2^ | 2550 | [11] |
| PPTA | 1.0 mA cm^−2^/1 mAh cm^−2^ | 2500 | [16] |
| USPH | 1.0 mA cm^−2^/1.0 mAh cm^−2^ | 600 | [18] |
| PAM-CS | 1.0 mA cm^−2^/1.0 mAh cm^−2^ | 1200 | [19] |
| ZnSO_4_-THF | 2.0 mA cm^−2^/2.0 mAh cm^−2^ | 1300 | [20] |
| CTAB-ZS | 1 mA cm^−2^/0.5 mAh cm^−2^ | 2500 | [21] |
| PDZ-H gel | 2.0 mA cm^−2^/4.0 mAh cm^−2^ | 1350 | [22] |
| This work | 1.0 mA cm^−2^/1.0 mAh cm^−2^ | 2600 |  |

**Table S4.** Cycling performance of the Zn||I_2_ batteries reported before.

| Material(s) | Current density  (A g^−1^) | Specific capacity (mAh g^−1^) | Capacity retention rate × Cycle number | Reference |
| --- | --- | --- | --- | --- |
| Zn\|\|NH_2_-MIL-125/GF\|\|I_2_@TiO_2_/NPC | 2 | 105.0 | The capacity retention rate was 88% after 10000 cycles | [23] |
| Zn\|\|PC-PVA/ZnCF_3_SO_3_ \|\|I_2_@AC | 1 | 155.2 | The capacity retention rate was 92.6% after 5000 cycles | [12] |
| Zn\|\|CSSA\|\|I_2_@KB | 0.5 | 195.5 | The capacity retention rate was about 96% after 1000 cycles | [24] |
| Zn\|\|PAM-MDMA \|\|I_2_@AC | 2 | 185.6 | The capacity retention rate was about 95% after 8000 cycles | [25] |
| Zn\|\|PZG\|\|I_2_@KB | 2 | 90.7 | The capacity retention rate was 96.4% after 10000 cycles | [6] |
| Zn\|\|PVA/Bmim[ZnBr_3_]\|\|I2@AC | 0.2 | About 120.0 | The capacity retention rate was about 96% after 4500 cycles | [17] |
| Zn\|\|PCA/ZnSO_4_\|\|I_2_@KB | 1 | About 210.0 | The capacity retention rate was about 97% after 1000 cycles | [26] |
| Zn\|\|TAMI/ZnSO_4_\|\|I_2_@PC | 0.5 | 196.9 | The capacity retention rate was about 95% after 50 cycles | [27] |
| Zn\|\|ELA/ZnSO_4_\|\|I_2_@KB | 1 | About 120.0 | The capacity retention rate was 79.39% after 2500 cycles | [28] |
| Zn\|\|NCNF\|\|I_2_@AC | 3 | 125.76 | The capacity retention rate was about 95% after 4000 cycles | [29] |
| This work | 2 | 145.5 | The capacity retention rate was about 97.4% after 10000 cycles |  |

**Reference:**

1.	G. Kresse, J. Furthmuller, "Efficient Iterative Schemes for Ab Initio Total-Energy Calculations Using a Plane-Wave Basis Set," *Physical Review B* 54, 16 (1996): 11169-11186, <https://doi.org/10.1103/PhysRevB.54.11169>.

2.	J. P. Perdew, K. Burke, M. Ernzerhof, "Generalized Gradient Approximation Made Simple," *Physical Review Letters* 77, 18 (1996): 3865-3868, https://doi.org/10.1103/PhysRevLett.77.3865.

3.	G. Kresse, D. Joubert, "From Ultrasoft Pseudopotentials to the Projector Augmented-Wave Method," *Physical Review B* 59, 3 (1999): 1758-1775, <https://doi.org/10.1103/PhysRevB.59.1758>.

4.	S. Grimme, J. Antony, S. Ehrlich, H. Krieg, "A Consistent and Accurate Ab Initio Parametrization of Density Functional Dispersion Correction (Dft-D) for the 94 Elements H-Pu," *Journal of Chemical Physics* 132, (2010): 154104, <https://doi.org/10.1063/1.3382344>.

5.	S. Grimme, S. Ehrlich, L. Goerigk, "Effect of the Damping Function in Dispersion Corrected Density Functional Theory," *Journal of Computational Chemistry* 32, (2011): 1456-1465, <https://doi.org/10.1002/jcc.21759>.

6.	X. Zhang, J. Y. Li, F. J. Xie, et al., "Polyzwitterionic Gel Electrolyte: Dual Optimization of Polyiodide Shuttle Suppression and Anode Stabilization in Aqueous Zn-I_2_ Batteries," *Advanced Functional Materials* 35, (2025): 2505132, <https://doi.org/10.1002/adfm.202505132>.

7.	Y. P. Yang, L. W. Shi, Y. Q. Wu, et al., "High Voltage, Toughness and Improved Interfacial Ion Deposition through a Dual-Crosslinked and Self-Healable Hydrogel Electrolyte for Green Zinc-Ion Batteries," *Chemical Engineering Journal* 484 (2024): 149780, <https://doi.org/10.1016/j.cej.2024.149780>.

8.	J. L. Yang, T. Xiao, T. Xiao, et al., "Cation-Conduction Dominated Hydrogels for Durable Zinc-Iodine Batteries," *Advanced Materials* 36, (2024): e2313610, <https://doi.org/10.1002/adma.202313610>.

9.	H. Xia, G. Xu, X. Cao, et al., "Single-Ion-Conducting Hydrogel Electrolytes Based on Slide-Ring Pseudo-Polyrotaxane for Ultralong-Cycling Flexible Zinc-Ion Batteries," *Advanced Materials* 35, (2023): e2301996, <https://doi.org/10.1002/adma.202301996>.

10.	J. L. Cong, X. Shen, Z. P. Wen, et al., "Ultra-Stable and Highly Reversible Aqueous Zinc Metal Anodes with High Preferred Orientation Deposition Achieved by a Polyanionic Hydrogel Electrolyte," *Energy Storage Materials* 35 (2021): 586-594, <https://doi.org/10.1016/j.ensm.2020.11.041>.

11.	J. Y. Zhao, X. L. Wang, B. Y. Tang, Y. F. Li, D. T. Liu, G. S. Zhu, "Amidoxime-Functionalized Hydrogel Electrolyte Enables Dendrite-Free and Shuttle-Free Zinc-Iodine Batteries," *Journal of Energy Chemistry* 114 (2026): 536-545, <https://doi.org/10.1016/j.jechem.2025.10.021>.

12.	Y. Xiong, H. R. Cheng, Y. K. Jiang, et al., "A Novel Water-Reducer-Based Hydrogel Electrolyte for Robust and Flexible Zn-I_2_ Battery," *Energy Storage Materials* 74 (2025): 103981, <https://doi.org/10.1016/j.ensm.2024.103981>.

13.	T. Y. Yang, T. T. Su, H. L. Wang, K. Li, W. F. Ren, R. C. Sun, "''Tennis Racket" Hydrogel Electrolytes to Synchronously Regulate Cathode and Anode of Zinc-Iodine Batteries," *Journal of Energy Chemistry* 102 (2025): 454-462, <https://doi.org/10.1016/j.jechem.2024.11.004>.

14.	Q. P. Jin, S. M. Gao, Y. C. Wang, Z. Y. Qin, "Synergistic Construction of Hierarchical Tree-Ring Structures and Blocked Interfaces for Stable, Flexible Zinc-Iodine Batteries with Ultrahigh Areal/Gravimetric Capacity," *Acs Sustainable Chemistry & Engineering* 12, (2024): 6744-16758, <https://doi.org/10.1021/acssuschemeng.4c06869>.

15.	R. M. Liu, D. D. Wang, Z. Gao, et al., "Low-Self-Discharge Nanoconfined Hydrogel Electrolyte for Stable High-Energy-Density Aqueous Zinc-Iodine Batteries," *Acs Nano* 20, (2026): 5217-5227, <https://doi.org/10.1021/acsnano.5c20669>.

16.	S. C. Cao, A. W. Zhang, H. Y. Fang, et al., "Skin-Like Quasi-Solid-State Electrolytes for Spontaneous Zinc-Ion Dehydration toward Ultra-Stable Zinc-Iodine Batteries," *Energy & Environmental Science* 18, (2025): 3395-3406, <https://doi.org/10.1039/d4ee05527f>.

17.	Z. Y. Hu, Z. X. Han, H. X. Liu, et al., "Mechanically Strong and Tough Ionic Liquid Gel Electrolyte for Four-Electron Zinc-Iodine Batteries," *Journal of the American Chemical Society* 147, (2025): 46632-46641, <https://doi.org/10.1021/jacs.5c18431>.

18.	Y. F. Wang, W. L. Yan, X. J. Zhu, et al., "Boosting Performance of Quasi-Solid-State Zinc Ion Batteries Via Zincophilic Solubilization," *Angewandte Chemie-International Edition* 64, (2025): e202508556, <https://doi.org/10.1002/anie.202508556>.

19.	D. H. Xu, Y. G. Wang, H. Tian, Y. Y. Chen, X. Z. Tian, Q. Y. Zhang, "Dynamic Cross-Linking Network Construction of Flexible Hydrogel Electrolyte Enabling Dendrite-Free Zinc Anode," *Advanced Energy Materials* 15, (2025): 2502217, <https://doi.org/10.1002/aenm.202502217>.

20.	T. Huang, Z. X. Wang, T. Z. Li, et al., "Multifunctional Phosphor with High-Efficient near-Infrared Emission Based on Antimony-Zinc Halides," *Acs Applied Materials & Interfaces* 16, (2024): 31322-31331, <https://doi.org/10.1021/acsami.4c04622>.

21.	Z. X. Liu, R. Wang, Y. C. Gao, et al., "Low-Cost Multi-Function Electrolyte Additive Enabling Highly Stable Interfacial Chemical Environment for Highly Reversible Aqueous Zinc Ion Batteries," *Advanced Functional Materials* 33, (2023): 2308463, <https://doi.org/10.1002/adfm.202308463>.

22.	H. Y. Lu, J. S. Hu, L. T. Wang, et al., "Multi-Component Crosslinked Hydrogel Electrolyte toward Dendrite-Free Aqueous Zn Ion Batteries with High Temperature Adaptability," *Advanced Functional Materials* 32, (2022): 2112540, <https://doi.org/10.1002/adfm.202112540>.

23.	L. Wang, J. P. Guan, N. Li, et al., "Amine-Functionalized Mil-125 Separator and Mof-Derived Carbon Host for High-Performance Aqueous Zinc-Iodine Batteries," *Advanced Energy Materials* 15, (2025): e04201, <https://doi.org/10.1002/aenm.202504201>.

24.	Y. B. Zhu, H. D. Zhang, M. Y. Tao, et al., "A Universal Janus Biopolymer Separator Enabled Dual-Interfacial Regulation toward Dendrite-Free and Shuttle-Free Zinc-Iodine Batteries," *Advanced Materials* 38, (2026): e72959, <https://doi.org/10.1002/adma.72959>.

25.	Y. Y. Wang, K. J. Zhong, J. K. Zhang, et al., "Concomitant Zinc Dendrite Mitigation and Iodide Shuttle Confinement: A Bifunctional Zwitterionic Hydrogel Electrolyte Unlocking Ultralong-Cycling Aqueous Zinc-Iodine Batteries," *Advanced Functional Materials* 36, (2026): e26937, <https://doi.org/10.1002/adfm.202526937>.

26.	F. F. Wang, W. B. Liang, X. Y. Liu, et al., "A Bifunctional Electrolyte Additive Features Preferential Coordination with Iodine toward Ultralong-Life Zinc-Iodine Batteries," *Advanced Energy Materials* 14 (2024): 2400110, <https://doi.org/10.1002/aenm.202400110>.

27.	J. M. Kang, J. J. Zhang, W. Wan, et al., "A Dynamic Cathode Interlayer for Ultralow Self-Discharge and High Iodide Utilization in Zinc-Iodine Batteries," *Energy & Environmental Science* 18, (2025): 4800-4810, <https://doi.org/10.1039/d4ee05584e>.

28.	Y. Jin, J. Cao, C. Huang, X. An, S. H. Wang, X. L. Yang, "Dual-Site Functional Orchestration Enables Synergistic Anodic Modulation and Cathodic Mooring for Durable Zinc-Iodine Batteries," *Nano-Micro Letters* 18, (2026): 301, <https://doi.org/10.1007/s40820-026-02144-5>.

29.	Y. M. Liu, X. F. Tian, L. Wang, et al., "Separator-Free Aqueous Zinc-Iodine Batteries: An in Situ Dual-Confinement Interface Enabling Electrostatic Reversible Polyiodide Capture/Release," *Energy & Environmental Science* 19, (2026): 2723-2734, <https://doi.org/10.1039/d6ee00296j>.
